# Cementless Allograft-Prosthetic Composite Reconstruction Using an Interlocking Revision Stem for Salvage Reverse Total Shoulder Arthroplasty: A Case Report

**DOI:** 10.7759/cureus.114651

**Published:** 2026-08-17

**Authors:** Swagato Kanjilal, Joseph V Marzano, Paul Ragusa

**Affiliations:** 1 Medical School, Lake Erie College of Osteopathic Medicine, Erie, USA; 2 Orthopaedic Surgery, St. John's Riverside Hospital, Yonkers, USA

**Keywords:** allograft prosthesis, cementless stem, major trauma, orthopaedic trauma surgery, orthopedic surgeon, proximal humerus fracture, reverse shoulder arthroplasty

## Abstract

Proximal humerus fractures can present in many ways and have many treatment options. The Neer classification is used to describe fractures to help guide treatment. However, treatment is often surgeon-dependent due to the multitude of variables in the proximal humerus: whether it be the rotator cuff, important arteries supplying blood to the proximal humerus, or varying functional goals between patients. Surgical approaches may include fracture fixation, hemiarthroplasty, and reverse total shoulder arthroplasty. We present the case of a 55-year-old man who underwent salvage reverse total shoulder arthroplasty with allograft-prosthetic composite reconstruction for a proximal humerus fracture nonunion. The procedure was performed using a long revision stem with interlocking screws. The purpose of this implant is to enable restoration of appropriate length and rotation during reconstruction without the need for cement. The patient was lost to follow-up after his six-week postoperative visit, but demonstrated significant improvement in range of motion and function. However, more research needs to be done as to what the best approach is for proximal humerus reconstruction.

## Introduction

Proximal humerus fractures are common injuries that frequently occur in young patients due to high-energy trauma and in elderly patients following low-energy, ground-level falls. Proximal humerus fractures require appropriate evaluation and management to restore normal shoulder function, which is necessary for performing daily activities. Treatment options range from non-operative management to surgical intervention. Surgical approaches may include fracture fixation, hemiarthroplasty, and reverse total shoulder arthroplasty, depending on the patient’s age, bone quality, and the specific fracture pattern [1]. This patient sustained a comminuted proximal humerus fracture from a slip and fall. After about six months, radiographs showed a proximal humerus nonunion. The patient eventually underwent salvage reverse total shoulder arthroplasty with allograft-prosthetic composite (APC) reconstruction for a proximal humerus fracture nonunion. The implant used allowed restoration of appropriate length and rotation during reconstruction without the need for cement, as required in conventional techniques [2]. Rather, interlocking screws were used to secure rotational and axial stability. This also allowed for intraoperative adjustment and trialing to maximize patient outcomes. 

## Case presentation

This report describes the case of a 55-year-old right-hand-dominant man with a past medical history significant for cerebrovascular accident with residual left-sided weakness, hypertension, and polysubstance use (cocaine). He sustained a slip-and-fall injury approximately six months prior to presentation. He was initially evaluated by another surgeon and diagnosed with a right proximal humerus fracture. Surgery was recommended; however, the patient was unable to proceed at that time due to ongoing treatment for cocaine use disorder.

He presented to us six months after the initial injury, following another fall that worsened his symptoms. On physical examination, there was swelling and tenderness over the proximal humerus, without scarring or evidence of muscle atrophy. Crepitus and motion were noted at the fracture site. The patient guarded the extremity, holding the arm in internal rotation with the forearm resting against the abdomen. Attempts at active or passive motion elicited significant pain; therefore, range of motion, strength, and provocative testing were deferred. Motor function and sensation of the axillary, musculocutaneous, anterior interosseous, posterior interosseous, ulnar, and radial nerves were intact. Radial pulses were normal.

**Figure 1:**
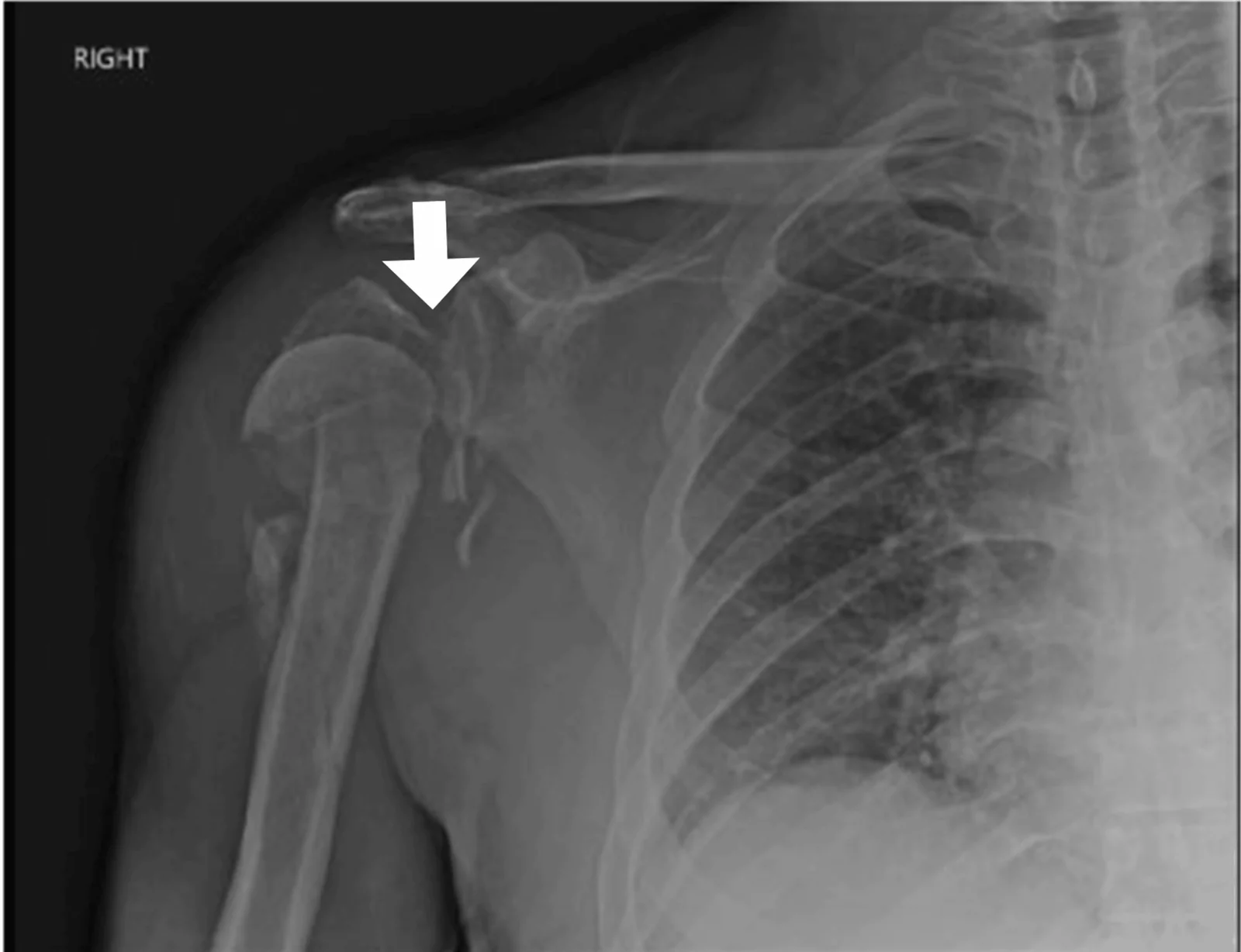
Right shoulder radiograph in internal rotation demonstrating severe comminution and malunion/nonunion of the proximal humerus White arrow points to the comminution of proximal humerus.

**Figure 2:**
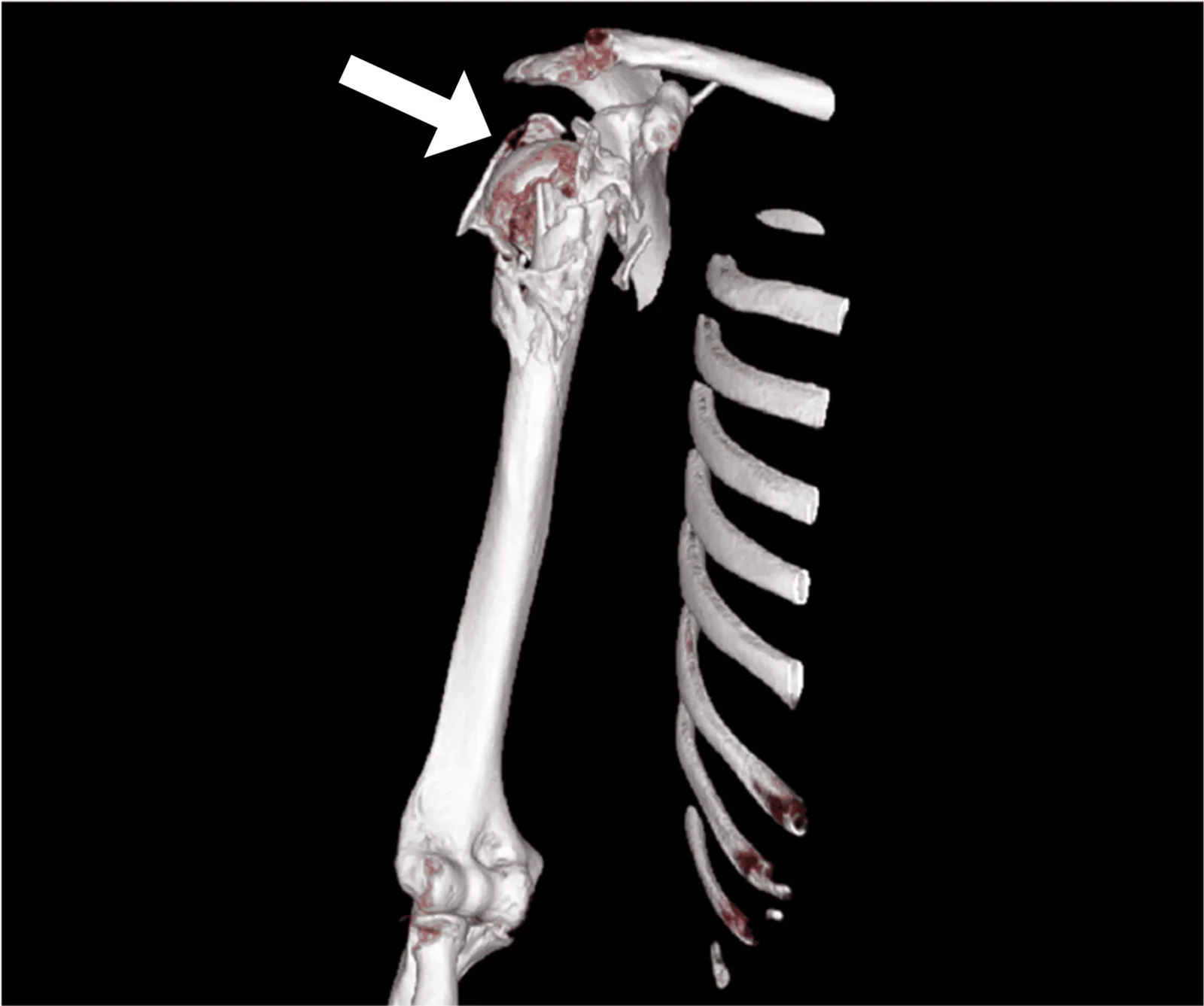
Three-dimensional visual of the patient’s right proximal humerus from the culmination of multiple views on CT demonstrating severe comminution and malunion/nonunion White arrow points to the comminuted fracture of the proximal humerus.

Radiographs and CT scan of the right upper extremity (without contrast) demonstrate an acute versus subacute fracture through the proximal one-third of the humeral shaft without displacement, and a severely comminuted and displaced subacute versus chronic fracture of the proximal humerus with multiple displaced fracture fragments (Figures 1, 2). There was hypertrophic callus formation. The patient was diagnosed with a right-sided four-part proximal humerus fracture nonunion as demonstrated in Figures 1, 2.

We discussed treatment options ranging from nonoperative measures to surgical options. We discussed surgical options ranging from fracture fixation to hemiarthroplasty to reverse total shoulder replacement. After discussion of risks and benefits of each approach, we agreed that an arthroplasty would be our procedure of choice in terms of maximizing their function with a single procedure. Fracture fixation has a high likelihood of failure to heal due to the degree of comminution and bone loss. We also discussed the possible need to use a proximal humerus structural APC depending on the quality of the bone and degree of bone loss. Surgery was eventually agreed upon due to the patient’s functional demands and his reliance on the right arm for daily activities due to left-sided weakness from a prior stroke. When setting up for the procedure, an interscalene block was administered, and the patient was positioned into a beach chair position. Antibiotics were given within 1 hour of the first surgical incision.

The procedure began with a deltopectoral incision. Once the proximal humerus was exposed, severe comminuted nonunion with extensive hypertrophic callus formation was revealed. All nonviable fragments were removed, and the remaining humeral shaft was prepared for reconstruction. The medullary canal was opened with a burr and hand reamers, which was confirmed with biplanar fluoroscopy. 

Next, the glenoid was prepared for a reverse total shoulder arthroplasty. After protecting the axillary nerve and excising the labrum, the glenoid was exposed. The inferior portion of the glenoid was reamed in preparation to place a 24 mm peg baseplate. Screws were then drilled for the 4.5 mm screws, screwing the central screw first, followed by the peripheral locking screws. The glenosphere was 40 mm with a 10-degree tilt and a 3.5 mm offset, which had enough lateral offset to be appropriate for the patient. We were unconcerned with the lateralization of the system due to the patient lacking risk factors for acromial stress fractures. Therefore, maintaining the deltoid angle and having adequate deltoid tension were prioritized. 

We then turned our attention to the humerus. Given the significant proximal humeral bone loss, a proximal humerus APC was selected. The allograft was carefully fashioned to reconstruct the proximal humerus and restore appropriate length, version, and offset. The APC was contoured to overlap the native humeral shaft, maximizing bony contact surface area to promote healing and incorporation. The APC was then assembled on the back table, and a V135 long stem prosthesis was implanted into the allograft, ensuring appropriate alignment and stability. The humeral canal was sequentially prepared, and the V135 long stem prosthesis-allograft composite was implanted. The stem was secured with interlocking screws to provide rotational and axial stability. The attached tendons on the allograft were incorporated into the surrounding soft tissues to assist with soft tissue balancing and reconstruction. Fluoroscopy was used intraoperatively to confirm appropriate implant position, length, alignment, and fixation. A +3 mm 135/145 polyethylene component was selected to change the humeral neck-shaft angle to 145 degrees, which was more stable for the new anatomy created by the reverse total shoulder arthroplasty. After the implantation, excellent range of motion was noted, and the tendons did not show excessive tension. The wound was irrigated and closed in a layered fashion. 

**Figure 3:**
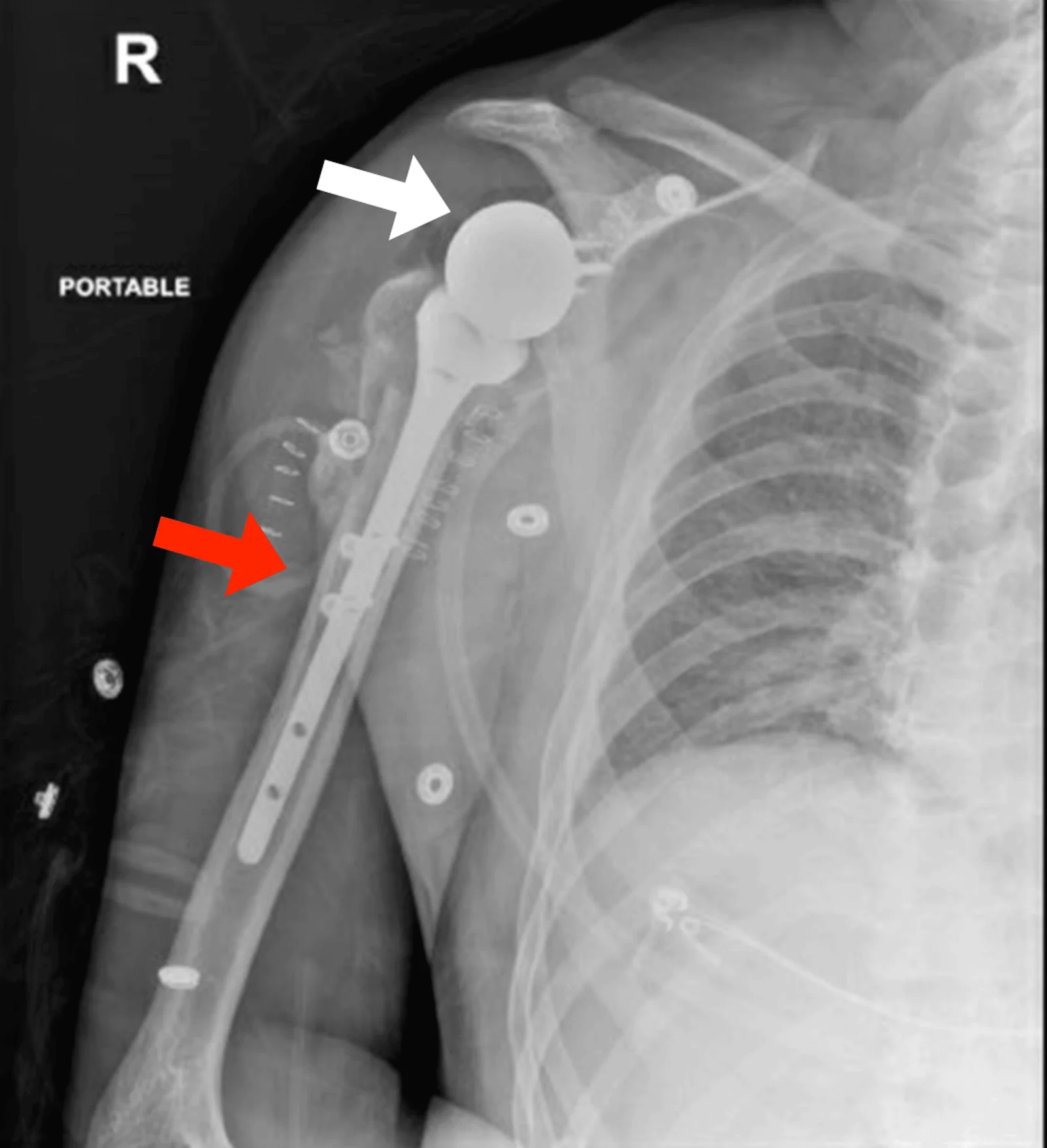
Right shoulder radiograph in internal rotation on postoperative day 0 demonstrating the allograft composite as well as the rTSA The white arrow points to the allograft and glenosphere. The red arrow points to the interlocking screws. rTSA, reverse total shoulder arthroplasty.

**Figure 4:**
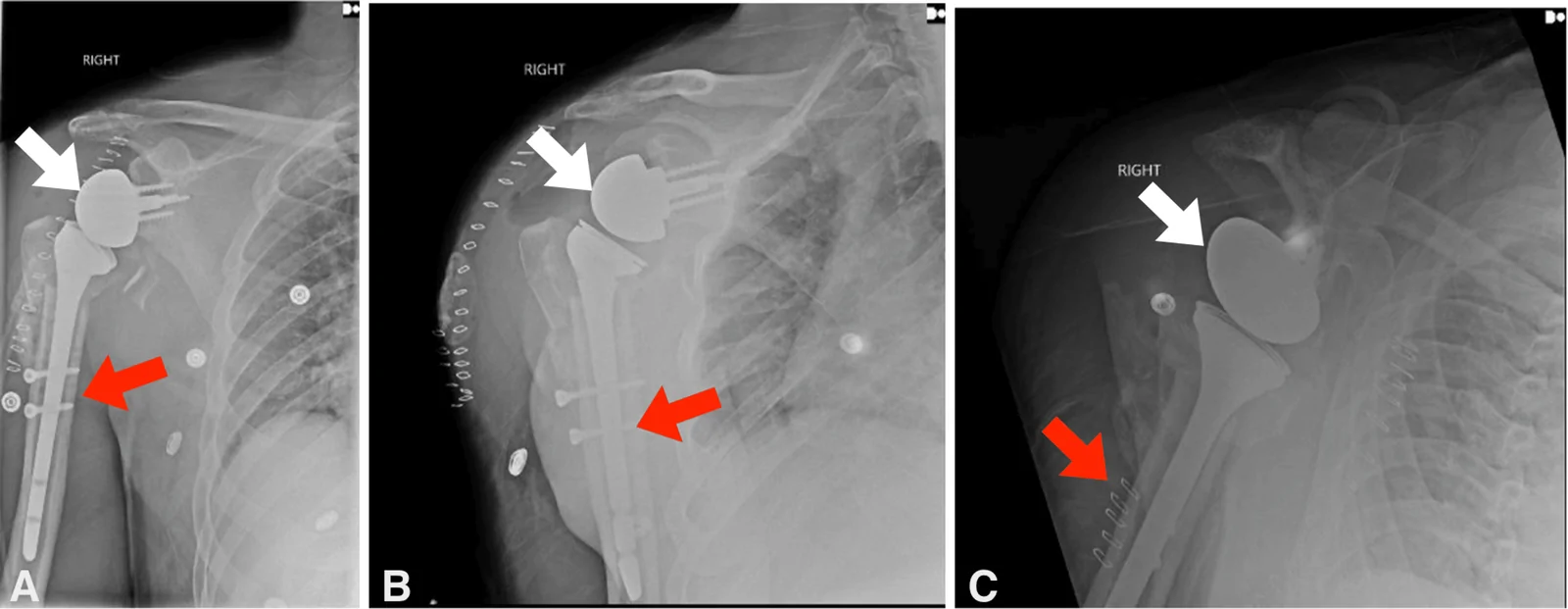
Right shoulder X-rays on postoperative day 2 demonstrating an allograft composite on an rTSA, including the allograft and glenosphere along with the interlocking screws A: Right shoulder AP X-ray. B: Right shoulder AP X-ray in internal rotation. C: Right shoulder scapular-Y X-ray. The white arrows point to the allograft and glenosphere. The red arrows point to the interlocking screws. AP, anteroposterior; rTSA, reverse total shoulder arthroplasty.

Postoperatively, the patient overall did well. Regular radiographs were taken to ensure stability of the prosthesis, evaluate for implant loosening, and assess incorporation of the allograft with the native bone (Figures 3, 4). The white and red arrows in Figures 3, 4 refer to the allograft and the distal interlocking screws, respectively. The patient suffered from mild postoperative fever, but the procedure was tolerated well, and the patient was discharged on postoperative day 11. The patient was encouraged to do physical therapy and delay weight bearing. On the most recent clinic visit at four weeks, the patient demonstrated the ability to actively externally rotate to 20 degrees and forward elevate to 60 degrees. The patient will continue to be followed regularly to ensure successful recovery. At the six-week follow-up visit, the patient’s forward flexion was 90 degrees, external rotation was 20 degrees, and internal rotation was to the back pocket. 

## Discussion

APCs are commonly used for reconstruction of the proximal humerus, particularly following tumor resection [3-5]. They are also used in cases involving significant proximal humeral bone loss secondary to trauma or infection [3-5]. One of the greatest technical challenges in chronic conditions such as nonunion or infection is accurately determining the appropriate implant height and rotation.

Several methods may assist in preoperative planning, including scaled full-length bilateral humerus radiographs to estimate the extent of bone loss. However, these measurements can still be unreliable due to soft tissue contracture and scarring commonly present in chronic nonunion and infection cases. In our experience, the most reliable method is intraoperative assessment of soft tissue tension. This can be achieved by inserting the humeral stem and telescoping the implant within the native humeral canal to determine the appropriate tension and positioning.

Once the optimal height and rotation are established, fixation of the humeral stem must be achieved. This process may require intraoperative adjustments and trial-and-error to optimize implant positioning. Traditionally, fixation is accomplished using cement in combination with cerclage wires and/or locking plates to secure the graft to native bone [3,4]. Current outcomes with the traditional allograft composite technique for the management of proximal humerus bone loss have shown mixed findings. Overall findings in the literature have shown high complication rates with fair functional outcomes. Complications include implant loosening, periprosthetic infection, and rotator cuff failure [6]. In contrast, the present case utilized a unique humeral implant with interlocking capabilities, allowing stable fixation at the desired height and rotation without the use of cement. This design also facilitated easier intraoperative adjustment and trialing of implant position. Achieving accurate implant height and rotation is critical for long-term implant success and functional outcomes.

The patient was lost to follow-up after the six-week postoperative visit. The patient demonstrated significant improvement in range of motion. However, this is a limitation in determining the efficacy of this novel implant. Long-term follow-up is necessary to ensure graft incorporation, long-term fixation, and functional durability.

In summary, there remains no clear consensus regarding the optimal treatment of proximal humeral fracture nonunion [5]. Management options include both nonoperative and operative treatment, with surgical treatment consisting of either open reduction and internal fixation or arthroplasty [7]. These cases are frequently associated with significant proximal bone loss, often necessitating APC reconstruction of the proximal humerus [4,5]. Current evidence remains inconclusive regarding the most effective reconstructive technique following proximal humeral bone loss [8]. Further research is therefore needed to better define the indications for the various reconstructive approaches. Additionally, as APC techniques continue to gain popularity, more studies are needed to determine the optimal fixation methods, surgical techniques, and postoperative protocols, including the role of interlocking fixation versus traditional cemented constructs, to improve patient outcomes [9].

## Conclusions

Proximal humerus fractures are very complex. There are many variables that interact with one another due to the important anatomical structures that exist in that area. Treatment is often within the discretion of the surgeon and patient: whether it be non-operative or a variety of operations such as ORIF and intramedullary nailing. In this case, an APC was fashioned to replace the unsalvageable nonunion comminuted fracture. Furthermore, an unorthodox approach was taken in the surgery by using interlocking nails to allow for proper rotation as well as to allow for intraoperative adjustment. More research needs to be done as to what the most effective treatment is for unsalvageable proximal humerus fractures and what technique is best for proximal humerus reconstruction using an allograft.
